# Persistent extensor strength deficit despite acceptable clinical outcomes after surgical treatment of patella fractures: a mid- to long-term follow-up study

**DOI:** 10.1007/s00402-026-06307-2

**Published:** 2026-04-17

**Authors:** Raşit Emin Dalaslan, Mücahid Osman Yücel, Sönmez Sağlam, Mehmet Arıcan, Zekeriya Okan Karaduman, İsmail Sav

**Affiliations:** Orthopedics and Traumatology Department, Medical Faculty of Duzce University, Düzce, Turkey

**Keywords:** Patella fracture, Quadriceps weakness, İsokinetic dynamometry, Functional outcomes

## Abstract

**Introduction:**

To evaluate mid- to long-term clinical outcomes together with objective isokinetic measurements of knee flexor and extensor muscle strength in patients who underwent surgical treatment for AO/OTA type C patella fractures, and to investigate the relationship between fracture severity, clinical scores, and strength deficits. We hypothesized that significant quadriceps strength deficits would persist despite acceptable clinical outcomes and that these deficits would be associated with worse pain and functional scores.

**Materials and methods:**

This retrospective cohort study included 58 patients treated surgically for patella fractures between 2015 and 2024, with a median follow-up duration of 60 months (IQR: 32–94; range: 13.1–132.0 months). The primary outcome was extensor muscle strength deficit measured by isokinetic dynamometry. Secondary outcomes included flexor strength deficit, Lysholm score, visual analog scale (VAS), and knee range of motion. Isokinetic dynamometry at 60°/s was used to measure concentric flexor and extensor peak torque values, and strength deficits were calculated by comparison with the contralateral limb. Outcomes were compared among fracture subtypes (C1, C2, C3), and correlations between strength deficits and clinical scores were analyzed. Non-parametric tests (Kruskal–Wallis) and partial correlation analysis (Pearson, adjusted for age) were used for statistical analysis.

**Results:**

At final follow-up, patients demonstrated generally acceptable clinical outcomes (mean Lysholm: 80.12 ± 16.88; mean VAS: 2.19 ± 1.79) with minimal extension deficits. However, the primary outcome, extensor muscle strength deficit, remained substantial a mean extensor strength deficit of 36.31 ± 17.27%, and 84.5% of patients exhibited marked extensor asymmetry (≥ 20%). Flexor strength deficits were less pronounced (19.09 ± 10.75%) but remained clinically relevant. No statistically significant differences were detected in clinical scores, range of motion, or strength deficits were observed among fracture subtypes (*p* > 0.05). Greater extensor and flexor strength deficits were significantly associated with higher VAS scores and lower Lysholm scores (all *p* < 0.05). Revision surgery was required in 12% of patients, and implant removal in 38%, indicating a considerable secondary surgical burden.

**Conclusions:**

Despite satisfactory clinical scores and radiographic healing, substantial and persistent quadriceps weakness appears to be common after surgically treated patella fractures. Objective strength deficits are strongly associated with pain and functional outcomes and may not be fully captured by conventional clinical assessments. Long-term management should therefore incorporate objective muscle strength evaluation and targeted rehabilitation strategies to address persistent extensor weakness.

**Supplementary Information:**

The online version contains supplementary material available at 10.1007/s00402-026-06307-2.

## Introduction

Patellar fractures account for approximately 1% of all skeletal injuries and frequently disrupt the extensor mechanism of the knee [1]. Although surgical fixation generally results in high union rates and satisfactory radiographic outcomes, persistent symptoms such as anterior knee pain, functional limitation, and extensor lag remain clinically relevant in the long term [2, 3].

Previous studies have primarily focused on patient-reported outcome measures (PROMs), including Lysholm scores, pain, and range of motion, which provide valuable but subjective assessments of recovery. However, objective evaluations have demonstrated that functional deficits may persist despite acceptable clinical scores. LeBrun et al. reported extension strength deficits of 26–31% at a mean follow-up of 6.5 years after operative treatment of patella fractures [3]. Similarly, other studies have shown persistent deficits in knee extensor strength, power, and endurance even after apparent structural healing [4].

These findings suggest that conventional clinical assessments may not fully reflect the functional capacity of the extensor mechanism. Supporting evidence from related knee pathologies, such as patellar dislocations, also indicates that quadriceps weakness may persist long term despite improvement in subjective outcomes [5].

Despite these observations, studies simultaneously evaluating both subjective clinical outcomes and objective muscle strength, particularly over extended follow-up periods, remain limited. In addition, the relationship between fracture severity and long-term functional and strength outcomes has not been fully clarified.

Therefore, the present study aimed to evaluate mid- to long-term clinical outcomes together with objective measurements of knee flexor and extensor muscle strength in patients who underwent surgical treatment for patella fractures. In addition, the relationship between fracture severity, clinical scores, and muscle strength deficits was investigated. It was hypothesized that significant quadriceps strength deficits would persist despite acceptable clinical outcomes and that these deficits would be associated with worse pain and functional scores.

## Materials and methods

### Study design and patient selection

This retrospective cohort study was conducted at a single tertiary referral center. Patients who underwent surgical treatment for AO/OTA type C patella fractures between January 2015 and December 2024 were screened for eligibility. A minimum follow-up duration of one year was required for inclusion. A total of 76 patients were initially identified during the study period.

**Fig. 1:**
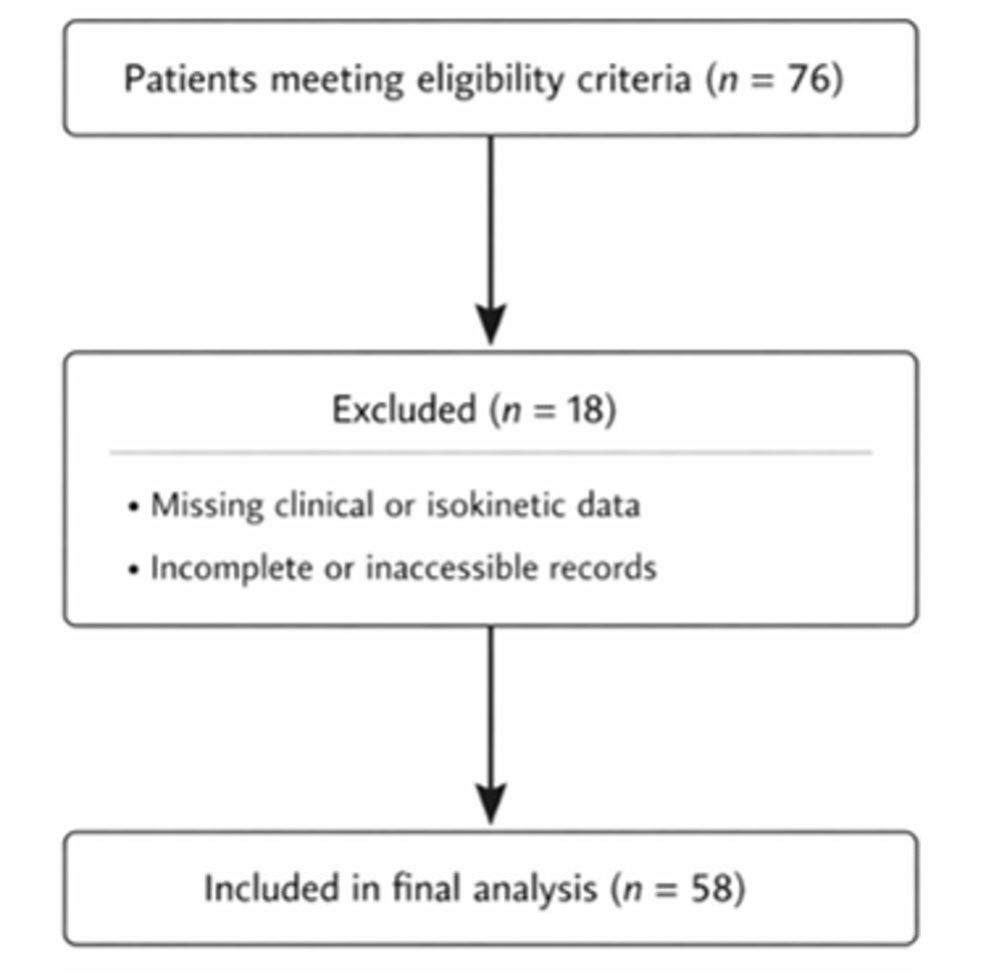
Flow diagram of patient selection and inclusion process

Patients younger than 18 years or older than 65 years, those with incomplete medical records, open fractures, concomitant neurovascular injuries, multitrauma, or a history of trauma, ligament injury, or previous surgery affecting the biomechanics of the contralateral knee were excluded from the study. Eighteen patients were excluded due to incomplete clinical or isokinetic data at final follow-up. After applying the inclusion and exclusion criteria, 58 patients were included in the final analysis (Fig. 1). The median follow-up duration was 60 months (IQR: 32–94 months; range: 13.1–132.0 months).

This retrospective study was approved by the XXX University Non-Interventional Health Research Ethics Committee (Approval No: 2025/179). Written informed consent was obtained from all patients. The study was conducted in accordance with the principles of the Declaration of Helsinki.

### Surgical technique and postoperative rehabilitation

All patients were treated using a standardized surgical protocol by the same surgical team. All fractures were treated using a standardized tension band wiring technique; however, minor technical variations in implant configuration were present based on surgeon preference and fracture characteristics. The goal of surgery was anatomical reduction of the articular surface and restoration of extensor mechanism continuity.

Postoperatively, the knee was immobilized in full extension using a brace. Antimicrobial prophylaxis was administered for 24 h following surgery.

Quadriceps isometric exercises were initiated on the first postoperative day. Partial weight bearing with crutches was allowed as tolerated. From the first postoperative month onward, gradual increases in knee flexion and extension were permitted by progressive adjustment of brace angles. The brace was discontinued at the end of the second postoperative month. Patients with insufficient range of motion at two months received an additional one-month supervised physiotherapy program.

### Isokinetic muscle strength assessment

**Fig. 2:**
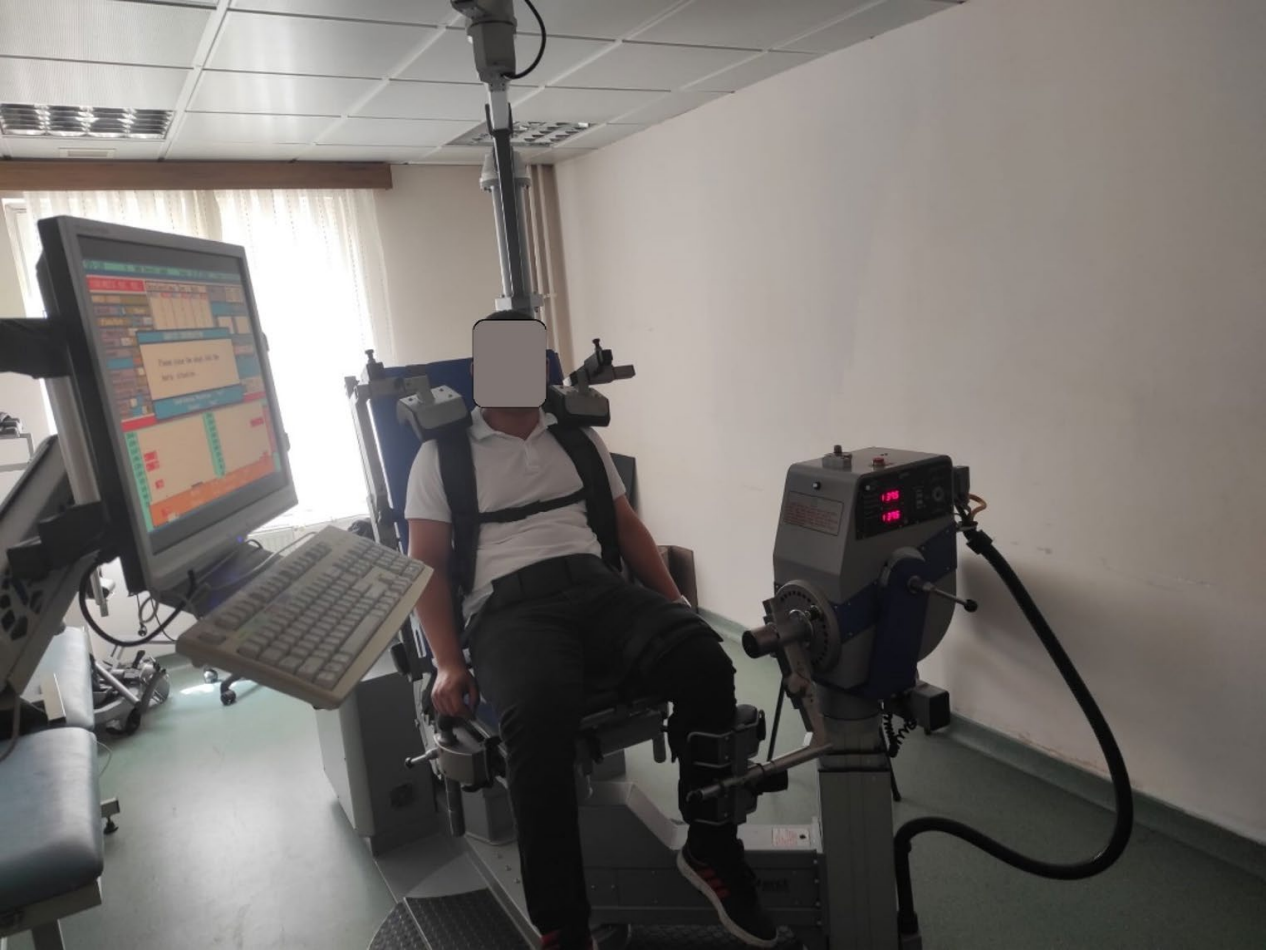
Isokinetic dynamometry setup for measuring knee flexor and extensor strength in the operated limb

Isokinetic muscle strength was evaluated using a validated and reliable isokinetic dynamometer (IsoMed 2000, D. & R. Ferstl GmbH, Hemau, Germany) [6](Fig. 2). Patients were seated with the hip flexed at 90°, and the trunk, pelvis, and thigh were stabilized with straps to minimize compensatory movements. The rotational axis of the dynamometer was carefully aligned with the anatomical axis of the knee joint, defined by a line through the femoral condyles. The lever arm was secured to the distal lower leg. After gravity correction and calibration, participants completed a standardized warm-up session. Concentric knee flexion and extension strength were measured at an angular velocity of 60°/s over an 80° range of motion. Each limb was tested separately, and peak torque values were recorded. Strength measurements of the operated limb were compared with the contralateral healthy limb [7].

Quadriceps muscle strength symmetry was evaluated by calculating the percentage difference in peak torque values between the operated and contralateral limbs, as measured by isokinetic dynamometry.

Based on commonly accepted criteria in the literature, a side-to-side difference of ≤ 10% was considered normal and is frequently used as a benchmark for return-to-sport clearance. A difference of 10–15% was defined as mild asymmetry, ≥ 15% as clinically significant muscle strength asymmetry, and ≥ 20% as marked pathological asymmetry [8, 9].

The percentage deficit was calculated using the following formula:

$$\begin{aligned}&{\text{Strength Deficit }}\left( \% \right) \\ & \quad ={\text{ }}[({\text{Contralateral Limb}}\, - \,{\text{Operated Limb}})/{\text{Contralateral Limb}}] \times {\mathrm{1}}00\end{aligned}$$

### Clinical evaluation

Clinical outcomes were assessed using the Lysholm Knee Scoring Scale, a widely used instrument evaluating eight domains: pain, swelling, instability, locking, limp, support, stair climbing, and squatting [10]. Although the Lysholm score was originally developed for ligament injuries, it is widely used in the assessment of knee function in various conditions, including patella fractures, and allows comparison with previous studies. The total score ranges from 0 to 100, with higher scores indicating better knee function. Lysholm scores were categorized as poor (< 65), fair (65–83), good (84–94), and excellent (95–100) [11]. Knee range of motion (ROM) was measured using a standard goniometer.

The primary outcome of the study was the extensor muscle strength deficit measured by isokinetic dynamometry. Secondary outcomes included flexor muscle strength deficit, Lysholm score, VAS score, and knee range of motion.

### Data collection

Demographic and clinical data were obtained from medical records, including age, sex, height, weight, mechanism of injury, comorbidities, complications, revision surgeries, and perioperative and postoperative radiographic findings; additionally, Lysholm scores, knee range of motion, and isokinetic flexor and extensor muscle strength values were recorded and compared between the operated and contralateral limbs.

### Statistical analysis

Descriptive statistics for continuous variables were presented as mean ± standard deviation (SD), median, minimum, maximum, and interquartile range (IQR), whereas categorical variables were expressed as frequencies and percentages. The normality of continuous variables was assessed using the Shapiro–Wilk test. Comparisons of continuous variables among fracture subtypes (C1, C2, and C3) were performed using the Kruskal–Wallis analysis of variance due to non-normal distribution. In addition, comparisons of percentage strength deficit values across clinical outcome categories were analyzed using the Kruskal–Wallis test. When a statistically significant difference was detected, Dunn’s multiple comparisons test was applied for post hoc analysis, and adjusted p-values were reported. The relationships between VAS and Lysholm scores and flexor and extensor strength deficit percentages were evaluated using partial correlation (Pearson adjusted for age) analysis, controlling for age. All statistical analyses were conducted using IBM SPSS Statistics for Windows, Version 20.0 (IBM Corp., Chicago, IL, USA), and a p-value < 0.05 was considered statistically significant.

## Results

A total of 58 patients were included in the study. The mean age was 46.43 ± 16.07 years, and 60.3% of the patients were male. The mean body mass index was 26.48 ± 4.51 kg/m². The mean length of hospital stay was 3.45 ± 2.12 days (Table 1).

The right knee was affected in 53.4% of patients. The most common mechanisms of injury were traffic accidents (44.8%) and simple falls (41.4%). According to the AO/OTA classification, 63.8% of fractures were type C1, 24.1% were type C2, and 12.1% were type C3. Revision surgery was required in 12.1% of patients. Complications were observed in 50% of patients, most commonly implant irritation requiring hardware removal (75.9% of complications), followed by nonunion requiring revision surgery (24.1%) (Table 1).

**Table 1:** Demographic and clinical characteristics of the patients

	Mean ± SD Median (Min–Max); (IQR)
Age (years)	46.43 ± 16.07 47 (18–65); (31–62)
BMI (kg/m²)	26.48 ± 4.51 26.16 (16.53–39.06); (23.37–28.29)
Sex, n (%)	
Female	23 (39.7)
Male	35 (60.3)
Fracture side, n (%)	
Right	31 (53.4)
Left	27 (46.6)
Mechanism of injury, n (%)	
Traffic accident	26 (44.8)
Simple fall	24 (41.4)
Fall from height	8 (13.8)
Fracture type	
C1	37 (63.8)
C2	14 (24.1)
C3	7 (12.1)
Length of hospital stay (days)	3.45 ± 2.12 3 (1–9); (2–4)
Revision surgery	
No	51 (87.9)
Yes	7 (12.1)
Complications	
No	29 (50.0)
Yes	29 (50.0)
Type of complications	
Implant irritation requiring hardware removal	22 (75.9)
Nonunion requiring revision surgery	7 (24.1)

The mean VAS score at final follow-up was 2.19 ± 1.79. The mean Lysholm score was 80.12 ± 16.88. The mean knee flexion was 117.07 ± 14.13°, and the mean extension deficit was 0.71 ± 2.05° (Table 2).

Isokinetic muscle strength evaluation revealed a mean flexor peak torque deficit of 19.09 ± 10.75% and a mean extensor peak torque deficit of 36.31 ± 17.27% compared to the contralateral limb (Table 2).

**Fig. 3:**
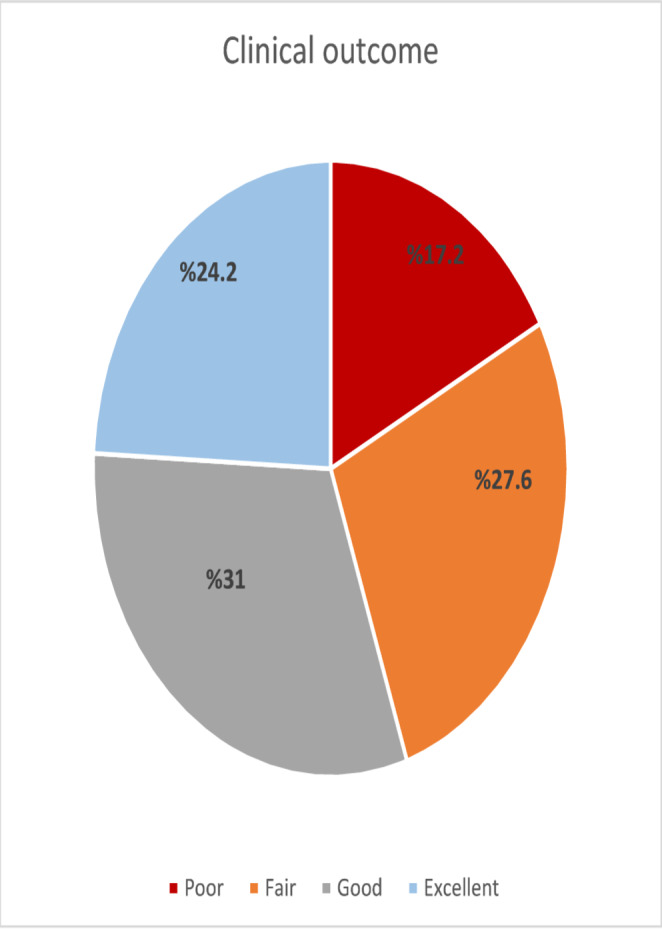
Distribution of patients according to Lysholm score categories (poor, fair, good, excellent) following surgical treatment of patella fractures

According to Lysholm score classification, 17.2% of patients had poor results, 27.6% fair, 31.0% good, and 24.1% excellent outcomes (Table 2)(Fig. 3).

**Table 2:** Clinical scores and muscle strength measurements of the patients

	Mean ± SD Median (Min–Max); (IQR)
VAS	2.19 ± 1.79 2 (0–8); (1–3)
Lysholm score	80.12 ± 16.88 84 (37–100); (65–94)
Flexion angle (°)	117.07 ± 14.13 120 (90–135); (108–130)
Extension angle (°)	0.71 ± 2.05 0 (0–10); (0–0)
Flexor peak torque deficit (%)	19.09 ± 10.75 17.1 (– 15.4 to 44.4); (14.3–25.1)
Extensor peak torque deficit (%)	36.31 ± 17.27 35.0 (– 9.4 to 73.6); (22.7–45.8)
Clinical outcome, n (%)	
Poor	10 (17.2)
Fair	16 (27.6)
Good	18 (31.0)
Excellent	14 (24.1)
Flexor strength deficit, n (%)	
Normal	5 (8.6)
Mild	13 (22.4)
Clinically significant	13 (22.4)
Marked	27 (46.6)
Extensor strength deficit, n (%)	
Normal	2 (3.4)
Mild	1 (1.7)
Clinically significant	6 (10.3)
Marked	49 (84.5)

**Fig. 4:**
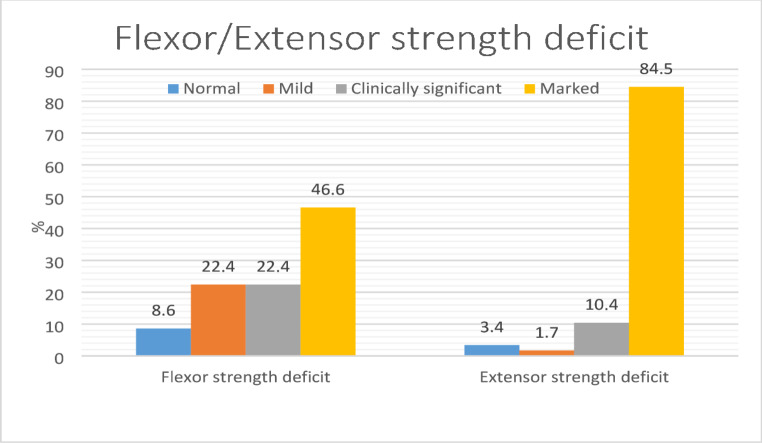
Distribution of flexor and extensor muscle strength asymmetry categories (normal, mild, clinically significant, and marked) in patients at final follow-up after surgical treatment of patella fractures

Regarding muscle strength asymmetry categories, flexor strength was normal in 8.6% of patients, mildly reduced in 22.4%, clinically significantly reduced in 22.4%, and markedly reduced in 46.6%. Extensor strength was normal in 3.4% of patients, mildly reduced in 1.7%, clinically significantly reduced in 10.3%, and markedly reduced in 84.5% of patients (Table 2) (Fig. 4).

**Table 3:** Comparison of VAS scores, Lysholm scores, flexion and extension angles, and flexor and extensor strength deficit percentages among patients with C1, C2, and C3 fracture types

	C1 fracture (*n* = 37)	C2 fracture (*n* = 14)	C3 fracture (*n* = 7)	*p* value
	Median (Min–Max) (IQR)	Median (Min–Max) (IQR)	Median (Min–Max) (IQR)	
VAS	2 (0–6); (1–3)	2 (0–8); (1–3)	3 (0–6); (1–4)	0.597 ^b^
Lysholm score	85 (37–100); (68–94)	80 (44–100); (61–96)	77 (5–95); (64–94)	0.667 ^b^
Flexion angle (°)	125 (90–135) (110–130)	117 (90–135) (98–126)	120 (90–130) (95–125)	0.380 ^b^
Extension angle (°)	0 (0–10) (0–0)	0 (0–10) (0–0.7)	0 (0–3) (0–0)	0.746 ^b^
Flexor peak torque deficit (%)	16.7 (–15.4 to 37.0) (14.6–23.3)	22.2 (3.8 to 44.4) (14.3–29.2)	24.2 (10.3 to 34.1) (12.5–32.7)	0.498 ^b^
Extensor peak torque deficit (%)	32.9 (–9.4 to 73.6) (22.2–46.6)	35.9 (11.8 to 69.7) (28.5–43.6)	44.6 (18.8 to 70.6) (29.8–51.9)	0.461 ^b^

^b^Kruskal–Wallis test

When clinical and functional outcomes were compared among fracture subtypes (C1, C2, and C3), no statistically significant differences were observed in VAS scores (*p* = 0.597), Lysholm scores (*p* = 0.667), knee flexion (*p* = 0.380), extension deficit (*p* = 0.746), flexor peak torque deficit (*p* = 0.498), or extensor peak torque deficit (*p* = 0.461). Although extensor strength deficit tended to be higher in C3 fractures, this difference did not reach statistical significance (Table 3).

**Table 4:** Comparison of flexor and extensor strength deficit percentages among patients with poor, fair, good, and excellent clinical outcomes

	Poor (*n* = 10)	Fair (*n* = 16)	Good (*n* = 18)	Excellent (*n* = 14)	*p* value
	Median (Min–Max) (IQR)	Median (Min–Max) (IQR)	Median (Min–Max) (IQR)	Median (Min–Max) (IQR)	
Flexor peak torque deficit (%)	25.3 (12.5 to 44.4) (21.7–33.7)	21.9 (3.8 to 36.4) (15.5–32.0)	15.4 (–15.4 to 26.4) (12.1–21.0)	14.7 (–13.3 to 29.9) (13.5–21.4)	**0.002** ^**b**^
Extensor peak torque deficit (%)	55.5 (44.9 to 70.6) (50.9–68.2)	35.9 (15.4 to 73.6) (29.9–47.2)	25.9 (–9.4 to 53.5) (19.7–33.8)	32.0 (11.8 to 44.6) (21.8–40.9)	**< 0.001** ^**b**^

^b^Kruskal–Wallis test, Statistically significant differences (*p* < 0.05) are presented in bold

A statistically significant difference was found in flexor peak torque deficit percentages among patients with poor, fair, good, and excellent clinical outcomes (*p* < 0.01) (Table 4). Post hoc pairwise comparisons were performed using Dunn’s multiple comparisons test to determine the source of the difference. No significant difference was observed between poor and fair outcomes (*p* = 1.000), poor and good outcomes showed a significant difference (*p* = 0.009), and poor and excellent outcomes also demonstrated a significant difference (*p* = 0.024). There were no significant differences between fair and good outcomes (*p* = 0.074), fair and excellent outcomes (*p* = 0.177), or good and excellent outcomes (*p* = 1.000).

A statistically significant difference was observed in extensor peak torque deficit percentages among patients with poor, fair, good, and excellent clinical outcomes (*p* < 0.001) (Table 4). Post hoc pairwise comparisons were performed using Dunn’s multiple comparisons test to identify the source of the difference. A significant difference was found between poor and fair outcomes (*p* = 0.041), poor and good outcomes (*p* < 0.001), and poor and excellent outcomes (*p* = 0.001). No significant differences were observed between fair and good outcomes (*p* = 0.241), fair and excellent outcomes (*p* = 1.000), or good and excellent outcomes (*p* = 1.000).

Overall, these findings indicate that significant differences in strength deficit percentages were primarily driven by patients with poor clinical outcomes, whereas no significant differences were observed among patients with fair, good, and excellent outcomes.

**Table 5:** Correlations between patients’ VAS and Lysholm scores and flexor and extensor strength deficit percentages

		Flexor deficit	Extensor deficit
VAS	r*	0.318	0.481
	p	0.016	< 0.001
Lysholm score	r*	–0.410	–0.615
	p	0.002	< 0.001

* Partial correlation coefficient (Pearson, adjusted for age)

Correlation analysis demonstrated that greater flexor and extensor strength deficits were significantly associated with higher VAS scores and lower Lysholm scores (all *p* < 0.05). The strongest association was observed between extensor strength deficit and Lysholm score (*r* = -0.615, *p* < 0.001) (Table 5).

## Discussion

In this mid- to long-term follow-up study of surgically treated patella fractures, substantial deficits in knee extensor strength were observed despite generally acceptable clinical outcomes. While most patients demonstrated satisfactory Lysholm scores and minimal extension deficits on physical examination, isokinetic testing revealed marked extensor strength deficits in the majority of patients. Flexor strength deficits were less pronounced but still present in a considerable proportion. Furthermore, greater extensor and flexor strength deficits were significantly associated with worse pain and functional outcomes, as reflected by higher VAS scores and lower Lysholm scores. Revision surgery and implant removal were observed in a notable proportion of patients, indicating a considerable secondary surgical burden. No statistically significant differences were detected among fracture subtypes; however, these subgroup analyses were exploratory and should be interpreted with caution. Overall, the findings of this study support the initial hypothesis that quadriceps strength deficits may persist despite acceptable clinical outcomes and may be associated with worse pain and functional scores.

Our findings are consistent with previous studies evaluating long-term functional outcomes after operatively treated patella fractures. LeBrun et al. reported persistent quadriceps strength, power, and endurance deficits at a mean follow-up of 6.5 years, with peak isometric extension deficits of 26–31% compared to the contralateral limb, despite generally acceptable clinical scores [3]. Similarly, a prospective cohort of 30 patients demonstrated marked deficits in knee extensor strength (–41%), power (–47%), and endurance (–34%) at one-year follow-up, despite complete fracture healing [4]. These observations align with our findings, where 84.5% of patients exhibited significant extensor strength deficits. High rates of symptomatic implant removal were also reported in both studies (37–52%), which mirrors our finding that 75.9% of complications were implant-related. In addition, a 15-year single-center retrospective cohort reported a 44.6% postoperative complication rate, with metalwork irritation being the most common cause of reoperation [12]. Furthermore, a nationwide study from the Swedish Fracture Register including 8726 patients demonstrated a significant decline in patient-reported functional outcomes one year after patella fracture, especially in operatively treated and complex AO/OTA fracture patterns [13]. Collectively, these studies indicate that persistent extensor weakness and the need for implant removal are common long-term issues, highlighting the importance of objective strength assessment and targeted rehabilitation, even when radiographic healing appears satisfactory.

The quadriceps deficits observed in our cohort are further supported by Biz et al., who systematically reviewed patients with primary or recurrent patellar dislocations [5]. Their review concluded that significant quadriceps femoris weakness frequently persists for up to three years, in both surgically and conservatively treated patients. This demonstrates that long-term extensor weakness is not exclusive to patella fractures but occurs in other patellar pathologies as well, emphasizing ongoing monitoring and rehabilitation of quadriceps strength.

Similarly, Girdwood et al. conducted a comprehensive longitudinal meta-analysis including 232 studies and over 34,000 patients, showing that knee extensor strength remains meaningfully reduced (> 10% compared with the contralateral limb and ~ 20% compared with uninjured controls) at one year following ACL reconstruction, with minimal improvement beyond 12–18 months and persistent deficits reported even beyond five years postoperatively [14]. These findings indicate that substantial and long-lasting quadriceps weakness may occur even after knee surgeries that do not directly involve the extensor mechanism, reinforcing the broader impact of knee trauma on neuromuscular function.

In a recent study on return-to-sport after open reduction and internal fixation (ORIF) for patella fractures, patients returned at a mean of 7 ± 3.9 months, with an overall high rate of 90.3%, yet only 51.6% resumed sports at or above pre-injury levels. Functional outcomes were not significantly associated with age or BMI [15]. Although return-to-sport was not directly measured in our cohort, the mean Lysholm score of 80.12 ± 16.88 and the finding that approximately half of patients achieved good or excellent outcomes suggest generally satisfactory function, while persistent extensor deficits may explain why many patients fail to regain pre-injury performance.

Quadriceps deficits after patellar fractures suggest that extensor weakness may not be solely attributable to direct mechanical disruption of the extensor mechanism. Similar impairments have also been reported following other knee injuries and surgical procedures, such as ACL reconstruction, which may indicate the involvement of broader neuromuscular mechanisms rather than purely localized structural damage [3, 5, 16]. Several studies have suggested that arthrogenic muscle inhibition (AMI), joint effusion, inflammation, and pain-related reflex inhibition could contribute to reduced voluntary quadriceps activation [17, 18]. In addition, cortical adaptations and alterations in motor control patterns have been proposed as potential mechanisms that may further exacerbate persistent weakness [19]. Therefore, it has been suggested that rehabilitation strategies might benefit from extending beyond general strengthening and range of motion exercises to include targeted quadriceps strengthening, neuromuscular re-education, and adjunctive interventions such as electrical stimulation aimed at reducing AMI and supporting long-term extensor recovery [5, 17].

Beyond functional impairment, quadriceps weakness may also influence pain progression. The Multicenter Osteoarthritis Study (MOST) showed that lower baseline quadriceps strength was associated with a higher risk of worsening knee pain in women, while no significant association was observed in men [20]. This suggests that persistent quadriceps weakness may contribute to long-term symptom progression. In our cohort, greater extensor strength deficits were significantly linked to higher VAS pain scores, supporting the idea that quadriceps weakness is not merely a mechanical deficit but also contributes to ongoing pain. Although our study did not specifically examine sex-related pain progression, the observed association aligns with evidence that inadequate quadriceps strength can predispose patients to sustained or worsening knee symptoms over time.

When examining the influence of fracture morphology on clinical and functional outcomes, literature shows mixed results. A recent retrospective study directly comparing simple (AO/OTA C1) and comminuted (AO/OTA C3) patellar fractures treated with tension band wiring found that C1 fractures had significantly higher Böstman functional scores than C3 fractures, suggesting that simpler fracture patterns may be associated with better functional outcomes [21]. At the same time, a clinical series evaluating multiple tension band wiring in AO/OTA C2 and C3 fractures demonstrated that the majority of patients achieved good to excellent functional results by 6 months, with significant improvement over time, indicating that even more complex fractures can recover well with stable fixation and early mobilization [22]. Although some studies suggest a trend toward better outcomes in less complex fractures, others report good functional recovery across C2–C3 patterns with appropriate management. In our study, we observed no statistically significant differences in mid- to long-term clinical or strength outcomes among C1, C2, and C3 fractures, suggesting that, over time, fracture severity alone may not be the primary determinant of functional recovery.

From a clinical perspective, the interpretation of functional scores should be considered in light of thresholds of clinical importance such as the patient acceptable symptom state (PASS). Although most patients in the present study demonstrated acceptable Lysholm scores, the substantial extensor strength deficits observed suggest that these patients may not fully reflect an optimal functional state. This discrepancy highlights that commonly used clinical scores may not fully reflect residual functional impairment, particularly in terms of objective muscle performance.

This study has several limitations that should be considered when interpreting the results. First, its retrospective design may introduce selection bias and limits the ability to establish causal relationships. Second, although isokinetic dynamometry provides objective measurements of muscle strength, pre-injury baseline strength values were not available for comparison, and contralateral limbs were used as controls. Third, due to the retrospective design of the study, no a priori sample size calculation was performed. In particular, the relatively small and unbalanced C2 and C3 subgroups may have limited the statistical power of between-group comparisons. Therefore, these subgroup analyses should be interpreted as exploratory, and the absence of statistically significant differences should not be considered evidence of equivalence. Furthermore, the associations between muscle strength deficits and clinical outcomes were assessed using partial correlation analysis adjusted only for age. Other potential confounding variables, including sex, BMI, and fracture type, were not simultaneously controlled for, and these findings should therefore be interpreted with caution. In addition, all patients were treated using tension band wiring, which may limit the generalizability of the findings to other fixation methods such as plate-based constructs or alternative techniques currently used in clinical practice. Rehabilitation adherence and specific physiotherapy protocols were not systematically monitored, which could have influenced the extent of muscle strength recovery. Although patients with prior knee pathology, previous surgery, or ligamentous injury were excluded to minimize baseline functional differences, pre-injury functional status could not be directly assessed. Therefore, the findings represent postoperative outcomes at final follow-up rather than changes from baseline.Sex-specific analyses were not performed due to the limited sample size and exploratory design of the study; therefore, potential sex-related differences could not be evaluated, which should be considered in accordance with SAGER recommendations. Finally, as this study was conducted at a single tertiary referral center, the generalizability of the findings to other settings or broader patient populations is limited.

## Conclusions

Despite generally acceptable clinical outcomes and minimal range of motion deficits, patients undergoing surgical treatment for patella fractures demonstrated persistent and often marked deficits in knee extensor strength at mid- to long-term follow-up. Extensor strength deficits were most pronounced in patients with poor clinical outcomes, and both flexor and extensor strength deficits were significantly correlated with higher pain levels and lower Lysholm scores. These findings underscore the importance of incorporating objective muscle strength assessment and targeted rehabilitation strategies in the long-term management of surgically treated patella fractures, as conventional clinical scores and radiographic healing may not fully reflect residual functional impairments.

## Supplementary Information

Below is the link to the electronic supplementary material.

Supplementary Material 1

## Data Availability

The datasets generated and/or analyzed during the current study are not publicly available due to institutional regulations and patient confidentiality but are available from the corresponding author on reasonable request.

## References

[1] Melvin JS, Mehta S (2011) Patellar fractures in adults. J Am Acad Orthop Surg 19:198–207. 10.5435/00124635-201104000-00004 21464213 10.5435/00124635-201104000-00004

[2] Büker N, Şavkin R (2022) Patella Kırıkları ve Rehabilitasyonu. Turkiye Klinikleri J Physiother Rehabil-Special Topics. Turkiye Klinikleri 8:60–69

[3] LeBrun CT, Langford JR, Sagi HC (2012) Functional outcomes after operatively treated patella fractures. J Orthop Trauma 26:422–426. 10.1097/BOT.0b013e318228c1a1 22183197 10.1097/BOT.0b013e318228c1a1

[4] Lazaro LE, Wellman DS, Sauro G, Pardee NC, Berkes MB, Little MTM et al (2013) Outcomes after operative fixation of complete articular patellar fractures: assessment of functional impairment. J Bone Joint Surg Am 95:e961–e968. 10.2106/JBJS.L.00012 10.2106/JBJS.L.00012 23864187

[5] Biz C, Nicoletti P, Agnoletto M, Bragazzi NL, Cerchiaro M, Belluzzi E et al (2024) Is there a strength deficit of the quadriceps femoris muscle in patients treated conservatively or surgically after primary or recurrent patellar dislocations? A systematic review and meta-analysis. J Clin Med 13:5288. 10.3390/jcm13175288 39274503 10.3390/jcm13175288 PMC11396229

[6] Mau-Moeller A, Gube M, Felser S, Feldhege F, Weippert M, Husmann F et al (2019) Intrarater reliability of muscle strength and hamstring to quadriceps strength imbalance ratios during concentric, isometric, and eccentric maximal voluntary contractions using the isoforce dynamometer. Clin J Sport Med 29:69–77. 10.1097/JSM.0000000000000493 28827499 10.1097/JSM.0000000000000493

[7] Hammami N, Bouzouraa E, Ölmez C, Hattabi S, Mhimdi N, Khezami MA et al (2024) Isokinetic knee strengthening impact on physical and functional performance, pain tolerance, and quality of life in overweight/obese women with patellofemoral pain syndrome. J Clin Med 13:4696. 10.3390/jcm13164696 39200838 10.3390/jcm13164696 PMC11355345

[8] Manoharan A, Fithian A, Xie V, Hartman K, Schairer W, Khan N (2024) Return to sports after anterior cruciate ligament reconstruction. Perm J 28:102–108. 10.7812/TPP/23.132 38659351 10.7812/TPP/23.132 PMC11232915

[9] Eagle SR, Keenan KA, Connaboy C, Wohleber M, Simonson A, Nindl BC (2019) Bilateral quadriceps strength asymmetry is associated with previous knee injury in military special tactics operators. J Strength Cond Res 33:89–94. 10.1519/JSC.0000000000002920 30431533 10.1519/JSC.0000000000002920

[10] Celik D, Coşkunsu D, Kiliçoğlu O (2013) Translation and cultural adaptation of the Turkish Lysholm knee scale: ease of use, validity, and reliability. Clin Orthop Relat Res 471:2602–2610. 10.1007/s11999-013-3046-z 23666590 10.1007/s11999-013-3046-z PMC3705057

[11] Wright RW (2009) Knee injury outcomes measures. J Am Acad Orthop Surg 17:31–39. 10.5435/00124635-200901000-00005 19136425 10.5435/00124635-200901000-00005

[12] Feathers JR, Fellows D, Richardson E, Khatir M, George A, Ashwood N (2025) Surgical outcomes following patella fracture repair: a single-center retrospective cohort study. Cureus 17:e92343. 10.7759/cureus.92343 41103849 10.7759/cureus.92343 PMC12522008

[13] Schmidt V, Rydberg EM, Krause M, Wolf O (2025) Patient-reported outcomes following patella fractures: a nationwide observational study of 8,726 patients from the Swedish Fracture Register. Bone Jt Open 6:1080–1089. 10.1302/2633-1462.69.BJO-2025-0141.R1 40925606 10.1302/2633-1462.69.BJO-2025-0141.R1 PMC12419904

[14] Tale of quadriceps and hamstring muscle strength after ACL reconstruction: a systematic review with longitudinal and multivariate meta-analysis - PubMed. https://pubmed.ncbi.nlm.nih.gov/39389762/. Accessed 27 Feb 2026 10.1136/bjsports-2023-107977 39389762

[15] Pesch S, Greve F, Zyskowski M, Müller M, Crönlein M, Biberthaler P et al (2023) High return to sports rates after operative treatment of patella fractures. Eur J Med Res 28:366. 10.1186/s40001-023-01359-1 37736742 10.1186/s40001-023-01359-1 PMC10514948

[16] Girdwood M, Culvenor AG, Rio EK, Patterson BE, Haberfield M, Couch J et al (2025) Tale of quadriceps and hamstring muscle strength after ACL reconstruction: a systematic review with longitudinal and multivariate meta-analysis. BMJ Publishing Group Ltd and British Association of Sport and Exercise Medicine. 10.1136/bjsports-2023-107977 10.1136/bjsports-2023-107977 39389762

[17] Rice DA, McNair PJ (2010) Quadriceps arthrogenic muscle inhibition: neural mechanisms and treatment perspectives. Semin Arthritis Rheum 40:250–266. 10.1016/j.semarthrit.2009.10.001 19954822 10.1016/j.semarthrit.2009.10.001

[18] Sonnery-Cottet B, Ripoll T, Cavaignac E (2024) Prevention of knee stiffness following ligament reconstruction: understanding the role of Arthrogenic Muscle Inhibition (AMI). Orthop Traumatol Surg Res 110:103784. 10.1016/j.otsr.2023.103784 38056774 10.1016/j.otsr.2023.103784

[19] Criss CR, Lepley AS, Onate JA, Clark BC, Simon JE, France CR et al (2023) Brain activity associated with quadriceps strength deficits after anterior cruciate ligament reconstruction. Sci Rep Nat Publishing Group 13:8043. 10.1038/s41598-023-34260-2 10.1038/s41598-023-34260-2 PMC10192374 37198275

[20] Glass NA, Torner JC, Frey Law LA, Wang K, Yang T, Nevitt MC et al (2013) The relationship between quadriceps muscle weakness and worsening of knee pain in the MOST cohort: a 5-year longitudinal study. Osteoarthritis Cartilage 21:1154–1159. 10.1016/j.joca.2013.05.016 23973125 10.1016/j.joca.2013.05.016 PMC3774035

[21] Büyükdoğan H, Sarıtaş TB, Yıldırım B, Ertürk C (2025) Comparison of functional outcomes of comminuted and simple patellar fractures treated with tension band wiring. Acta Med Alanya Alanya Alaaddin Keykubat Üniversitesi 9:179–184. 10.30565/medalanya.1712713

[22] Srivastava DP, Das DR, Singh DDK, Baghel DP (2026) Evaluatıon of functıonal outcome of commınuted patella fracture managed by multıple tensıon band wırıng ın ao type 34 C2 & C3. J Contemp Clin Pract 12:118–125. 10.61336/jccp/25-06-123

