# Supplementary material for: Persistent extensor strength deficit despite acceptable clinical outcomes after surgical treatment of patella fractures: a mid- to long-term follow-up study
Source: Arch Orthop Trauma Surg. 2026 Apr 17;146(1):147. doi: 10.1007/s00402-026-06307-2 (PMC13090276; doi:10.1007/s00402-026-06307-2)
Supplement: Supplementary file 1 — Supplementary Material 1 [file 402_2026_6307_MOESM1_ESM.doc]

STROBE Statement—Checklist of items that should be included in reports of ***cohort studies***

|  | Item No | Recommendation |
| --- | --- | --- |
| **Title and abstract** | 1 | (*a*) Yes – stated as “retrospective cohort study” in Abstract (Page 1) |
|  |  | (*b*) Yes – Abstract provides objectives, methods, results, and conclusions (Page 1) |
| Introduction | | |
| Background/rationale | 2 | Provided in Introduction (Pages 1–2) |
| Objectives | 3 | Clearly stated with hypothesis at end of Introduction (Page 2) |
| Methods | | |
| Study design | 4 | Described as retrospective cohort study (Methods section, Page 2) |
| Setting | 5 | Study center, timeframe (2015–2024), and follow-up described (Page 2) |
| Participants | 6 | (*a*) Inclusion/exclusion criteria and selection process described (Page 2) |
|  |  | (*b*) Not applicable |
| Variables | 7 | Outcomes (extensor/flexor strength deficits, Lysholm, VAS, ROM), predictors, and fracture classification defined (Page 2-3) |
| Data sources/ measurement | 8* | Isokinetic dynamometry and clinical scores described (Page 4-7) |
| Bias | 9 | Potential sources of bias (retrospective design, selection bias) addressed in Discussion (Page 10) |
| Study size | 10 | No a priori sample size calculation due to retrospective design (Methods & Discussion, Page 2,10) |
| Quantitative variables | 11 | Continuous variables and categorizations (e.g., strength deficit thresholds) explained (Page 3) |
| Statistical methods | 12 | (*a*) Statistical methods described (Kruskal–Wallis, partial correlation – Pearson adjusted for age) (Page 4) |
|  |  | (*b*) Subgroup analyses (C1–C3) described (Page 4) |
|  |  | (*c*) Patients with incomplete data excluded (Page 2) |
|  |  | (*d*) Not applicable |
|  |  | (*e*) Not performed |
| Results | | |
| Participants | 13* | (a) Flow described (76 screened, 18 excluded, 58 included) (Page 2) |
|  |  | (b) Reasons for exclusion provided (Page 2) |
|  |  | (c) Flow diagram included (Figure 1) |
| Descriptive data | 14* | (a) Patient characteristics reported (Page 4-5) |
|  |  | (b) No missing data in final cohort; patients with incomplete data were excluded (Methods, Page 2) |
|  |  | (c) Follow-up time: Median, IQR, and range reported (Page 2) |
| Outcome data | 15* | Outcome data: Clinical scores and strength deficits reported (Page 5-8) |
| Main results | 16 | (*a*) Unadjusted results (means, SDs, p-values) and age-adjusted partial correlation analyses are reported. Confidence intervals were not calculated due to the exploratory design (Results, Page 5-8). |
|  |  | (*b*) Category boundaries for muscle strength deficit percentages are defined in the Methods section (Page 3). |
|  |  | (*c*) Not applicable |
| Other analyses | 17 | Other analyses: Subgroup analyses and correlation analyses reported (Page 7-8) |
| Discussion | | |
| Key results | 18 | Key results: Summarized at beginning of Discussion (Page 8-9) |
| Limitations | 19 | Limitations: Clearly discussed (retrospective design, sample size, confounders, etc.) (Page 10) |
| Interpretation | 20 | Interpretation: Cautious interpretation provided (Page 10) |
| Generalisability | 21 | Generalisability: Addressed (single-center limitation) (Page 10) |
| Other information | | |
| Funding | 22 | Funding: no funding |

*Give information separately for exposed and unexposed groups.
